# DERM12345: A Large, Multisource Dermatoscopic Skin Lesion Dataset with 40 Subclasses

**DOI:** 10.1038/s41597-024-04104-3

**Published:** 2024-11-28

**Authors:** Abdurrahim Yilmaz, Sirin Pekcan Yasar, Gulsum Gencoglan, Burak Temelkuran

**Affiliations:** 1 Imperial College London, Division of Systems Medicine, Department of Metabolism, Digestion, and Reproduction, London, SW7 2AZ United Kingdom; 2 The University of Health Sciences, Haydarpasa Numune Research and Training Hospital, Department of Dermatology and Venereology, Istanbul, 34668 Turkey; 3 Istinye University, Liv Hospital Vadistanbul, Department of Dermatology and Venereology, Istanbul, 34010 Turkey

**Keywords:** Skin cancer, Basal cell carcinoma, Melanoma, Squamous cell carcinoma, Medical imaging

## Abstract

Skin lesion datasets provide essential information for understanding various skin conditions and developing effective diagnostic tools. They aid the artificial intelligence-based early detection of skin cancer, facilitate treatment planning, and contribute to medical education and research. Published large datasets have partially coverage the subclassifications of the skin lesions. This limitation highlights the need for more expansive and varied datasets to reduce false predictions and help improve the failure analysis for skin lesions. This study presents a diverse dataset comprising 12,345 dermatoscopic images with 40 subclasses of skin lesions, collected in Turkiye, which comprises different skin types in the transition zone between Europe and Asia. Each subgroup contains high-resolution images and expert annotations, providing a strong and reliable basis for future research. The detailed analysis of each subgroup provided in this study facilitates targeted research endeavors and enhances the depth of understanding regarding the skin lesions. This dataset distinguishes itself through a diverse structure with its 5 super classes, 15 main classes, 40 subclasses and 12,345 high-resolution dermatoscopic images.

## Background & Summary

Dermatoscopy, also known as epiluminescence microscopy, is the inspection of the skin using magnifying lenses and polarized or non-polarized filtered illumination, allowing examination of lesions invisible to the naked eye. It is the most extensively used and standardized diagnostic procedure in clinics to diagnose skin lesions. Various types of dermatoscopes are actively used in daily clinics. Hand-held dermatoscopes, for example, have become widely available, affordable, and widely employed, especially in developing countries. However, dermatologists’ diagnostic performance in dermatoscopic skin lesion identification is closely linked to their training and prior clinical experience, highlighting a variability inherent in human decisions. The prevalence and structure of malignant and benign lesions that vary depending on race, geographical factors and skin type also affects dermatologists’ performance. Addressing this gap, artificial intelligence (AI) trained on specialized dataset can reduce the experience gap that results from the human factor during the clinical examination of these lesions^1^.

One of the initial studies was published on the classification of only three classes: melanoma, nevus, and seborrheic keratosis^2,3^. The skin lesion dataset HAM10000, which had seven classes and common lesions, was published in 2018^4^. Eight classes with the squamous cell carcinoma skin lesion which is a common lesion but uncovered in HAM10000 was later presented by the BCN20000 dataset^5^. Among dermatoscopic skin lesion datasets, BCN20000 has the largest number of multi-class images (eight classes). New datasets continue to be published in open-access repositories such as The International Skin Imaging Collaboration (ISIC), which currently has over 485,000 public images (accessed on 25 Nov 2024)^6^. Enlarging open-access datasets supports the development of more robust and intelligent AI algorithms.

Datasets on skin lesions can be listed in four different categories: clinical, pathological slide, and dermatoscopic image datasets, and datasets combining more than one of these modalities. Clinical image datasets such as the Interactive Atlas of Dermatoscopy and the Dermofit Image Library can be accessible for a fee^7,8^. The MED-NODE Dataset^9^, the Asan and Hallym Dataset^10^, the SD-198/SD-260 Datasets^11–13^, PAD-UFES-20^14^, SCIN (5.2% skin lesion)^15^, Atlas Dermatologica^16^, DermaAmin^17^, and The SLICE-3D dataset with over 400,000 images (1,218 images with diagnoses)^18^ are all open-access. Fitzpatrick 17k Dataset is a dataset sourced from Atlas Dermatologica and DermaAmin, with the addition of Fitzpatrick skin type information (28.62% skin lesion)^19^. Diverse dermatology images (DDI) dataset also presents skin images with their skin type^20^. Cancer Genome Atlas is a pathological slide image dataset for skin lesions in the literature^21^. Clinical, dermatoscopic, and pathological images can all be found on DermNet NZ for free (high-resolution images for a fee)^22^. Study results show that the diagnostic accuracy (by clinicians and AI) achievable using dermatoscopic image datasets result in higher success rates when compared to those achievable using clinical image datasets, bringing out the importance of the dermatoscopic image datasets^23^. Pathological images require tissue excision from the patient. For that reason, pathological image dataset is not as practical as dermatoscopic observation.

PH^2^ dataset was published for benchmarking dermatoscopic images including with three lesion classes (common nevus, atypical nevus, and melanoma (mel))^24^. The Derm7pt dataset with five lesion classes (basal cell carcinoma (bcc), mel, miscellaneous, nevus (nv), and seborrheic keratosis (sk)), 2000 dermatoscopic and clinical images was published along with the 7-point checklist pattern analysis and its automation by using artificial neural networks^25^. The largest dermatoscopic dataset included in the scientific studies is the International Skin Imaging Collaboration (ISIC) archive with over 1,156,000 total images (accessed at: 25 Nov 2024). This archive contains various datasets such as: ISIC-2016 with 1,279 images and two lesion classes (benign and malignant)^2^, ISIC-2017 with 2,000 images three lesion classes (mel, nv, and sk)^3^, ISIC-2018^26^, HAM10000, with 10,015 images and seven lesion classes (akiec, bcc, benign-keratosis like (bkl), dermatofibroma (df), mel, nv, and vascular (vasc))^4^, BCN20000 with 18,946 images and eight lesion classes (actinic keratosis (ak), bcc, df, mel, nv, squamous cell carcinoma (scc), sk, and vasc)^5^, Patient-Centric dataset with 33,126 images and two lesion classes (benign and mel)^27^, and PROVe-AI dataset with 603 images and 15 lesion classes. Lastly, the dataset collected in Argentina with 10 subclasses (ak, bcc, df, mel, nv, scc, sk, solar lentigo (sl), lichenoid keratosis (lk), and vasc) was added into ISIC dataset^28^. Between 2016 and 2024, numerous competitions were held on challenges such as lesion segmentation, feature extraction, and lesion classification, using these ISIC datasets. The largest open access datasets that contain significant dermatoscopic images were shown in Table 1. 

These large datasets are crucial for the development of AI based models for skin lesion classification purposes. In addition, the introduction of subclass annotations in skin image datasets has high potential to enhance studies in the AI field focusing on skin disease, for creation of more trustworthy, robust, and intelligent systems. Here, our study presents a taxonomic tree for skin lesion classes and a dataset of 40 skin lesions annotated with subclasses for the first time in the scientific literature. Our comprehensive dataset, which contains a total of 12,345 images, was collected from Turkiye, where various skin types are encountered^29–31^. This dataset is one of the largest collections to date in terms of the total number of images in multiclass datasets.

## Methods

**Fig. 1:**
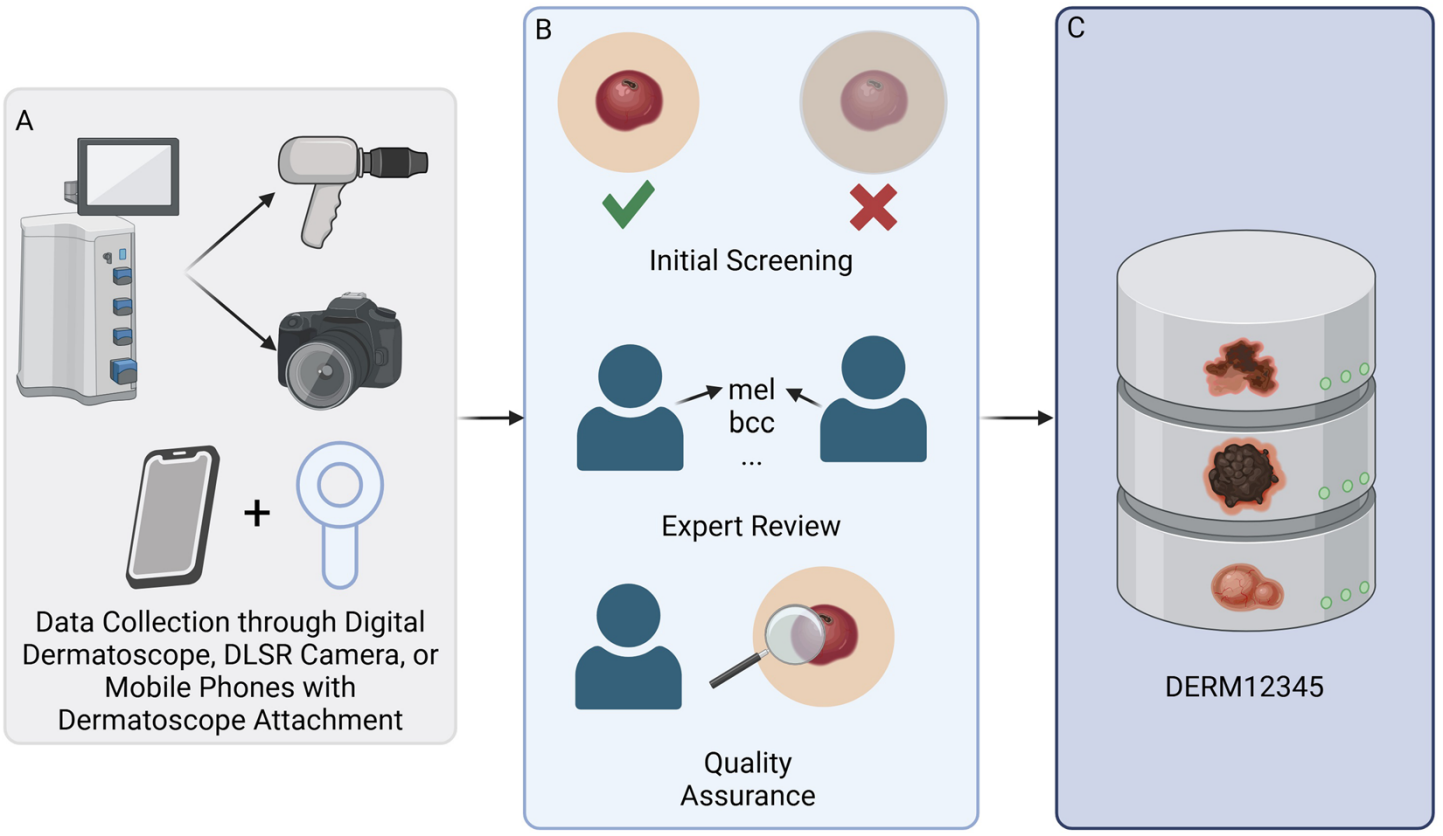
An overview of this study. (**A**) Skin lesion images are collected using a digital dermatoscope, DSLR camera, or mobile phones with dermatoscope attachment. (**B**) The collected images undergo an initial screening  based on inclusion and exclusion criterias, followed by the expert annotation (histopathology, follow-up, or consensus) and a final quality assurance step. (**C**) Schematic representation of the skin lesion dataset^50^.

The DERM12345 dataset contains 12,345 high-resolution dermatoscopic images from 1,627 patients, which were collected from 2008 to 2021 in the Department of Dermatology and Venereology at Celal Bayar University (Manisa, Türkiye), Istinye University (Istanbul, Türkiye) and University of Health Sciences Haydarpasa Numune Research and Training Hospital (Istanbul, Türkiye) by using MoleMax 3, MoleMax HD (Derma Medical Systems, Vienna, Austria), FotoFinder® videodermatoscope (FotoFinder Systems, Bad Birnbach, Germany) and 3gen DermLite DL4 hand-held dermatoscopes (DermLite LLC, California, United States of America) with connection kit for iPhone 5 and 7 (Apple Inc., California, United States of America). Ethical approval of images was based on ethics review board protocols 20.478.486/1023 (Manisa Celal Bayar University, 24/11/2021). The informed consent was waived because of the retrospective nature of the study as the dataset contains anonymized data. The final dataset includes 40 skin lesion classes. Figure 1 shows how the dataset constructed. The collection process includes capturing a wide variety of skin lesions using both digital dermatoscopy devices, high-resolution digital single-lens reflex (DSLR) cameras, or mobile phones with dermatoscope attachment. After the data collection, each images were initially screened in terms of image quality, magnification, and resolution. Then, the image were reviewed, annotated, and quality assurance ensured by two expert dermatologists.

### Data collection from digital dermatoscopy devices

Digital dermatoscopy devices are computer-attached systems and outfitted with dermatoscopes that utilize polarized light, enabling a magnification such as from 10x to 140x. This feature facilitates the thorough examination of deeper skin structures with a high level of detail. These dermatoscopy devices are linked to a computer system equipped with specialized software for the purpose of capturing and storing images. By using this software, dermatologists can follow up on the patients and their skin lesions with detailed information such as location and lesion classes. Each lesion is captured using a standardized technique and consistent lighting conditions to minimize any potential variability. Dermatoscopy is performed in the contact-polarized mode using an interface medium to avoid reflection caused by excessive scales, with ultrasound gel applied when necessary. The dermatologists ensure the most possible and accurate alignment of the field of view, with the lesion at the center and a small margin of surrounding healthy skin included to provide contextual information. To generate our dataset, we exported the data using their software tools in suitable formats such as HTML files. The raw data was subsequently retrieved from the raw files as lesion images and their metadata information. The cases were selected from this system by two expert dermatologists according to their consensus benign diagnosis, follow-up, and excised lesions with a histopathologic report.

### Data collection from mobile phones and hand-held dermatoscopes

Mobile phone integrated dermatoscopes are low-cost and accessible devices that can provide state-of-art image quality. In this dataset, Apple iPhone 5 and 7, equipped with a 3gen DermLite DL 4 hand-held dermatoscope kits, were used to collect dermatoscopic images. The DermLite DL4 attachment with mobile devices enables the utilization of polarized light dermatoscopy, which offers a maximum magnification of 10x. The use of high-level magnification is essential for thoroughly analyzing the intricate components of the skin. The mobile phone was utilized alongside dermatoscope attachments, facilitating the acquisition of high-resolution (such as at 3840 × 2160 pixels, 4K resolution) skin lesion images directly onto the phone. The mobile images, along with their metadata, including lesion classes, were systematically documented, and matched with their records on digital dermatoscopy devices. A standardized image acquisition process was adhered to keep consistent lighting and framing conditions for all images. The dermatologists conducted the imaging by positioning the lesions in the center of the field of view as possible while also including a border of healthy skin to offer contextual information. When focusing or centering the mobile phone camera was challenging, the dermatologists took images in different angles. This usually caused the edges of the dermatoscopes to appear as black regions. Images with relatively large black regions were cropped to increase the standardization. Those with smaller black regions were left intact to avoid cropping out the parts of the image with lesion information.

### Data selection and quality control

The raw dataset included not only dermatoscopic images but also non-dermatoscopic images and other images such as device test images. To accelerate the data selection procedure, the dermatoscopic images were extracted by utilizing a script in Python (version: 3.11.5) that selected the images together with their metadata information. The BeautifulSoup library (version: 4.12.2) was utilized for the automated extraction and categorization of images in HTML format. Data that could not be extracted or exported automatically in HTML format was hand-labeled and classified. The metadata was additionally stored manually in comma-separated values (CSV) files. To remove duplicate images, their file sizes were compared by using a script coded in Python. These scripts found 280 duplicate images. Then, they were manually reviewed. Consequently, all dermatoscopic images were retrieved and their corresponding information was stored in a CSV file. After automated extraction of the images, the careful selection of data was facilitated to maintain the integrity and usefulness of the dataset for both clinical reference and computational purposes. The criteria for selection were determined prior to the initiation of data collection and were consistently followed during the selection procedure.

**Table 1:** Specifications of the largest open access dermatoscopic skin lesion datasets in the literature.

Dataset	Study	Type	Dataset Size	Class Size
*PH*^2^	^24^	Dermatoscopic	200	3
Derm7pt	^25^	Dermatoscopic + Clinic	2,000	5
ISIC 2016	^2,48^	Dermatoscopic	100	3
ISIC 2017	^3^	Dermatoscopic	2,750	3
ISIC 2018 - HAM10000	^4,26^	Dermatoscopic	10,015	7
ISIC 2019	^4,5^	Dermatoscopic	25,331	8
ISIC 2020	^27^	Dermatoscopic	33,126	2
BCN20000	^5^	Dermatoscopic	18,946	8
Argentina	^28^	Dermatoscopic + Clinic	1,616	10
PROVe-AI	^49^	Dermatoscopic	603	15
DERM12345	Our	Dermatoscopic	12,345	40

### Inclusion Criteria

- **Image Quality:** Images must be clear, in focus, and have sufficient quality. All images were reviewed manually to ensure that key diagnostic features of the skin lesions are understandable.
- **Diagnostic Confirmation:** Only images of lesions with a consensus of clinical diagnosis by two expert dermatologists, a follow-up or a histopathological confirmation (where available) were included.

### Exclusion criteria

- **Poor Image Quality:** Images that were blurred, under- or over-exposed, or had large artifacts. All images were reviewed manually, and the images that could potentially interfere with diagnosis were excluded.
- **Incomplete Data:** Images lacking essential metadata such as the presumed diagnosis were not included in the dataset.
- **Ethical Concerns:** Images that contained identifiable patient information or did not have proper consent were excluded to uphold ethical standards.
- **Unsuitable:** Images which contain unsuitable skin diseases or artefacts were excluded but not limited to these classes: nail lesions, mucosal lesions, cockade nevus, leukoplakia, sarcoidosis psoriasis, verruca, cyst, keloid, sebaceous hyperplasia, wart, genital wart, lipoid proteinosis, subcorneal hemorrhage, pigmented purpuric dermatoses, mucosal melanosis, and some types of adenocarcinoma such as sebaceous carcinoma and eccrine glands.

### Selection process

The selection process involved multiple stages:

- **Initial Screening:** A preliminary review of images was conducted by trained engineers to remove images that obviously did not meet the inclusion and exclusion criteria. If one more than different classes of skin lesion is located in an image, these images were cropped.
- **Expert Review:** Two expert dermatologists then reviewed the remaining images to ensure that they met the clinical standards and diagnostic requirements.
- **Quality Assurance:** A final review was conducted by a panel comprising two dermatologists (Gulsum Gencoglan (GG) and Sirin Pekcan Yasar (SPY) with over 20 years experience on dermatoscopy) to ensure the images were of suitable quality for both clinical reference and computational tasks.

### Taxonomy tree

**Fig. 2:**
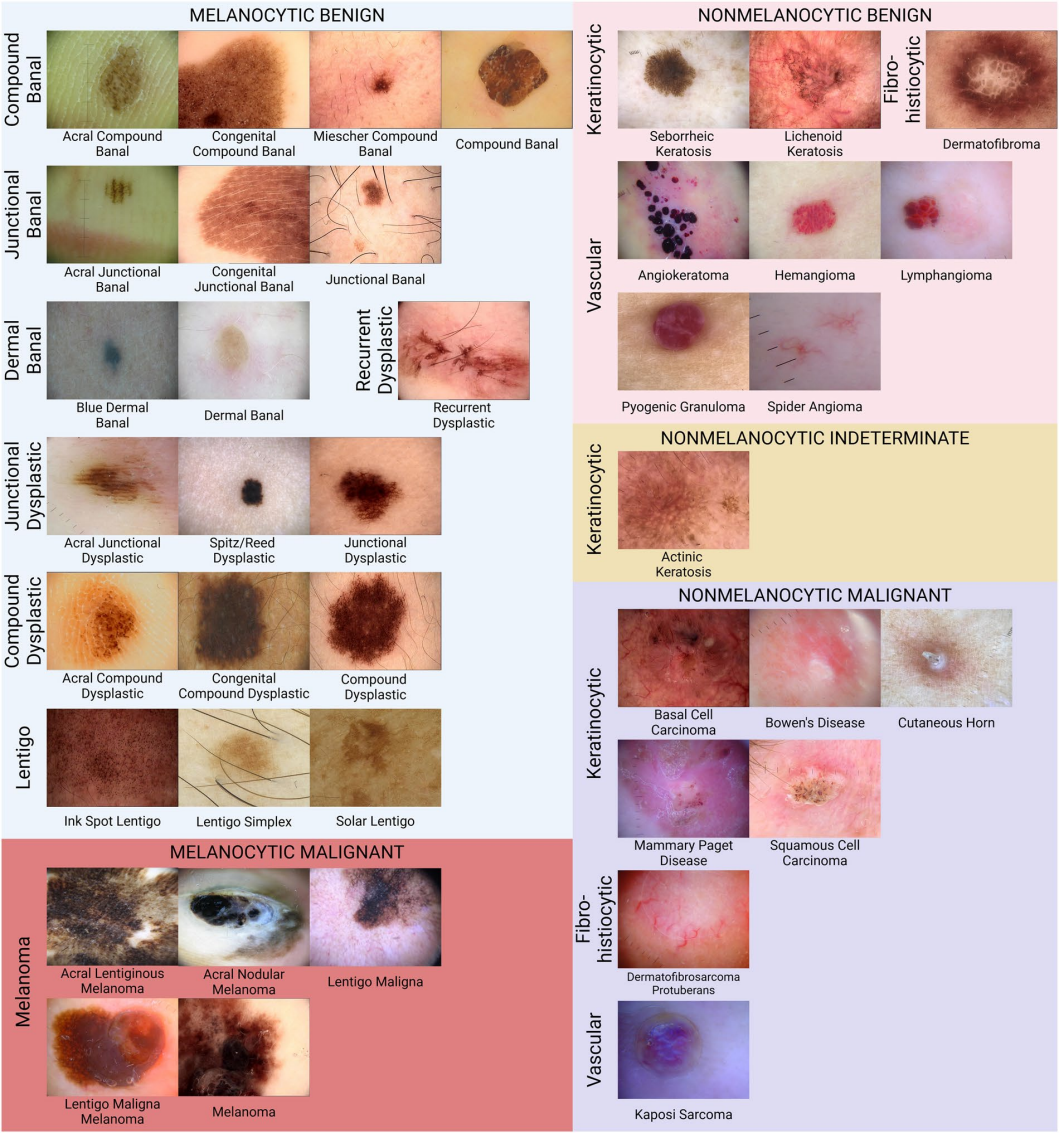
A representative image from each subclass^51^.

**Fig. 3:**
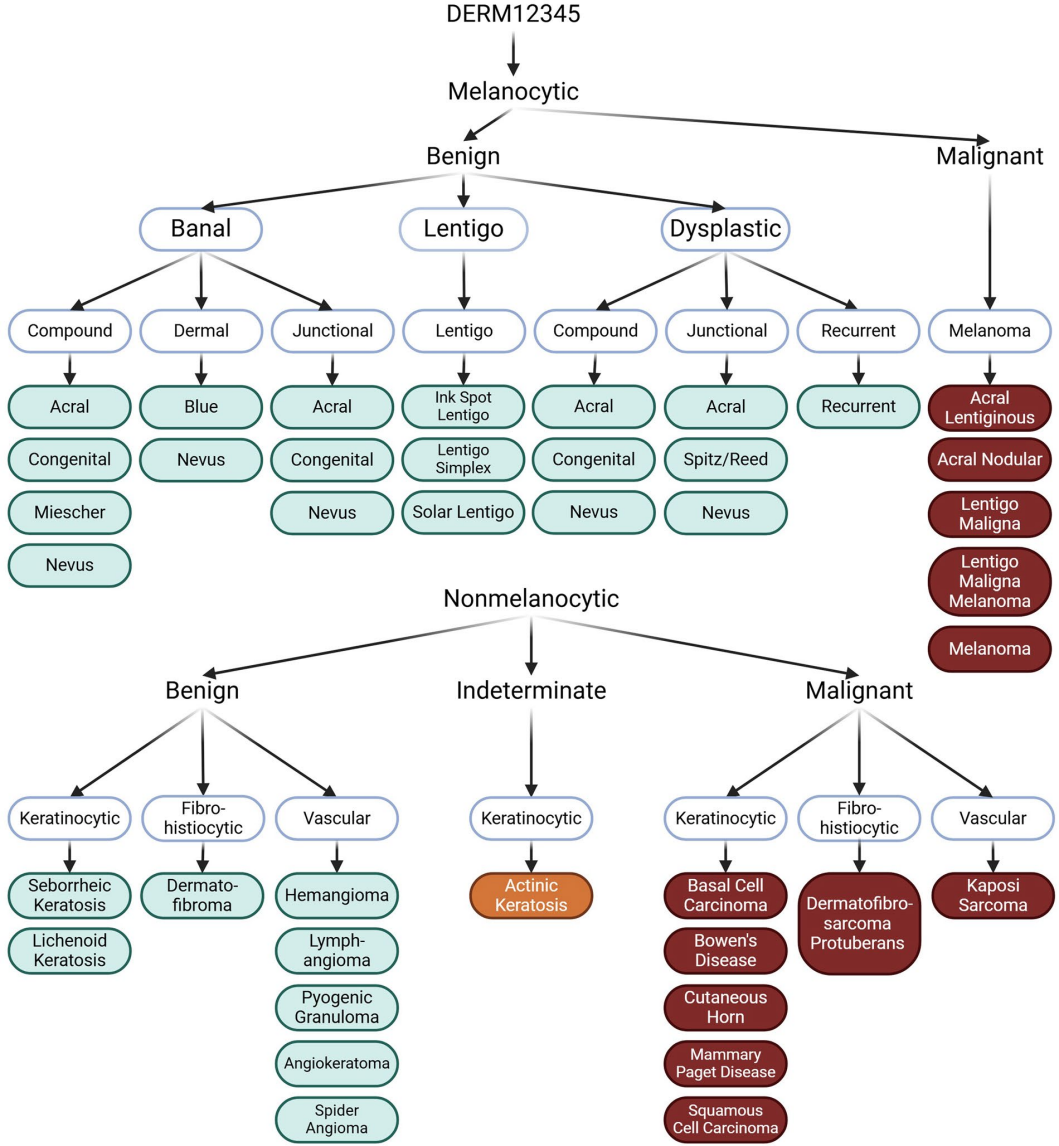
An overview of the taxonomy tree. The first level includes melanocytic and nonmelanocytic with benign, indeterminate, and malignant groups. The second level comprises banal, dysplastic and lentigo for melanocytic benign; melanoma for melanocytic malignant; keratinocytic, fibro-histiocytic, and vascular for nonmelanocytic benign; keratinocytic for nonmelanocytic-indeterminate; and keratinocytic, fibro-histiocytic, and vascular for nonmelanocytic malignant. The third level represents the subclasses associated with their respective main and super classes. Our taxonomy tree consists of 5 super classes, 15 main classes, and 40 subclasses^52^.

A three-level taxonomy tree and the dataset following the latest dermatological classification standards^32–34^ was created to guide the use of DERM12345. The benign skin lesions that are high risk of being or becoming malignant were especially classified into a separate subclass such as dysplastic nevus^35^, congenital nevus^36^, blue nevus^37^ and recurrent nevus that have the risk of unremoved malignant cell. The skin lesions were classified according to their specific anatomical localizations such as palm-sole (acral), face and trunk-extremities. Combined lesions with multiple diagnoses were excluded if cropping into separate images is not possible. Skin lesions were initially grouped into the two classes as melanocytic and non-melanocytic. These two classes were further categorized into malignant, indeterminate, and benign groups, and five super classes were created. These five super classes were then classified into 15 main classes. Lastly, sub-lesion types were then classified into 40 different subclasses. Melanocytic-benign lesions were classified into banal with compound, dermal, and junctional mainclasses, lentigo with ink spot lentigo (isl), lentigo simplex (ls), and solar lentigo (sl) subclasses, and dysplastic with compound, junctional, and recurrent (rd) mainclasses. Dysplastic dermal nevus were added into banal dermal nevus because of their similar patterns. Common nevus subclasses (acral, blue, Miescher, congenital, and Spitz/Reed nevus) under dermal, compound, and junctional groups were then classified into their subclasses if available. Melanocytic malignant was classified into five subclasses as acral lentiginous melanoma (alm), acral nodular melanoma (anm), lentigo maligna (lm), lentigo maligna melanoma (lmm), and melanoma (mel). Nonmelanocytic-benign lesions were classified into three mainclasses as keratinocytes (subclasses: lichenoid keratosis (lk), seborrheic keratosis (sk)), fibro-histiocytic (subclasses: dermatofibroma (df)), and vascular (subclasses: angiokeratoma (angk), hemangioma (ha), lymphangioma (la), pyogenic granuloma (pg), and spider angioma (sa)). Nonmelanocytic-malignant lesions were classified into three mainclasses as keratinocytes (subclasses: basal cell carcinoma (bcc), Bowen’s disease (bd), cutaneous horn (ch), mammary Paget disease (mpd), and squamous cell carcinoma (scc)), fibro-histiocytic (subclasses: dermatofibrosarcoma protuberans (dfsp)), and vascular (subclasses: Kaposi sarcoma (ks)). In addition, actinic keratosis (ak) was classified under nonmelanocytic-indeterminate subclasses. Figure 2 shows a sample image of each subclass. The taxonomy tree of these classes with their sizes were shown in Figure 3 and Table 2, respectively.

**Table 2:** The DERM12345 dataset contains 12,345 skin lesions categorized into 5 super classes, 15 main classes, and 40 subclasses.

Superclass	Mainclass	Subclass (Labels)	# of Images
Melanocytic Benign (10043 Images)	Banal Compound (1300 Images)	Acral (acb)	37
		Congenital (ccb)	418
		Miescher (mcb)	123
		Compound Nevus (cb)	722
	Banal Dermal (723 Images)	Blue (bdb)	163
		Dermal Nevus (db)	560
	Banal Junctional (1792 Images)	Acral (ajb)	426
		Congenital (cjb)	143
		Junctional Nevus (jb)	1223
	Lentigo (91 Images)	Ink Spot Lentigo (isl)	6
		Lentigo Simplex (ls)	25
		Solar Lentigo (sl)	60
	Dysplastic Compound (539 Images)	Acral (acd)	9
		Congenital (ccd)	32
		Compound Nevus (cd)	498
	Dysplastic Junctional (5582 Images)	Acral (ajd)	288
		Spitz-Reed (srjd)	19
		Junctional Nevus (jd)	5275
	Dysplastic Recurrent (16 Images)	Recurrent (rd)	16
Melanocytic Malignant (400 Images)	Melanoma (400 Images)	Acral Lentiginous (alm)	60
		Acral Nodular (anm)	76
		Lentigo Maligna (lm)	86
		Lentigo Maligna Melanom (lmm)	25
		Melanoma (mel)	153
Nonmelanocytic Benign (1077 Images)	Keratinocytic (615 Images)	Seborrheic Keratosis (sk)	607
		Lichenoid Keratosis (lk)	8
	Fibro-histiocytic (180 Images)	Dermatofibroma (df)	180
	Vascular (282 Images)	Angiokeratoma (angk)	39
		Hemangioma (ha)	166
		Lymphangioma (la)	56
		Pyogenic Granuloma (pg)	7
		Spider Angioma (sa)	14
Nonmelanocytic Indeterminate (58 Images)	Keratinocytic (58 Images)	Actinic Keratosis (ak)	58
Nonmelanocytic Malignant (767 Images)	Keratinocytic (747 Images)	Basal Cell Carcinoma (bcc)	423
		Bowen’s Disease (bd)	37
		Cutaneous Horn (ch)	12
		Mammary Paget Disease (mpd)	9
		Squamous Cell Carcinoma (scc)	266
	Fibro-histiocytic (4 Images)	Dermatofibrosarcoma Protuberans (dfsp)	4
	Vascular (16 Images)	Kaposi Sarcoma (ks)	16
Total			12,345

## Data Records

The dataset is uploaded to the Harvard Dataverse^38^ and is accessible at the following link 10.7910/DVN/DAXZ7P. It was released under a Creative Commons Attribution (CC BY) license. The images and metadata are accessible from this repository. This dataset will also be accessible from the ISIC archive that provides an advanced user and command line interface (CLI) for researchers. All images are in Joint Photography Expert Group (JPEG)^39^ format with their corresponding labels in metadata. The metadata file was generated in CSV file for easy processing. The metadata contains file names and lesion classes with their detailed taxonomic identification. The dataset was splitted into training (9860 images, 80%) and test (2485 images, 20%) sets based on patients to guide researchers for standardized benchmarks. Class balancing was considered during the partitioning process to ensure that each set is representative of the overall dataset. For subclasses with low sample sizes, the images were manually labeled (as training, and test) based on unique lesions to prevent data leakage (excluding dfsp and mpd, as there are few images of a single lesion in our dataset for these rare diseases). The subclass names of dysplastic and banal nevus are standardized by expressing them in acronyms using the initials of their subclasses and mainclasses such as (a)cral (j)unctional (b)anal nevus: ajb. Other subclasses are standardized by expressing them in acronyms using their initial letters such as (b)asal (c)ell (c)arcinoma: bcc.

### Dataset metadata

The dataset metadata contains this information:

- **Unique Image ID:** Displays the unique image identification number of an image.
- **Patient ID:** Displays the unique patient identification number of the image.
- **Image Type:** Identifies the modality of the image.
- **Copyright License:** Specifies the copyright license for the image.
- **Split:** Indicates which of the training or test dataset the image belongs to.
- **Super Class:** Shows the first-level category of the image.
- **Malignancy:** States whether the condition shown in the image is malignant or not.
- **Main Class 1:** Shows the second-level category of the image.
- **Main Class 2:** Shows the second-level category of the image.
- **Sub Class:** Shows the third-level category of the image.
- **Label:** Shows the label of the image.

## Technical Validation

All images in this dataset were of patients applying to the dermatology department with skin complaints. All lesions were classified and reviewed according to the rules of dermatoscopic evaluation by two expert dermatologists in a consensus decision. All malignant skin lesions were biopsy proven. The majority of these lesions consisted of follow-up images and were labeled using patient records. Nevus with over 2 years of digital dermatoscopic follow-up were annotated with no change except nevoid involution. The number of dysplastic nevus (N: 6,136) constituted a large part of the dataset. These dysplastic nevi were followed up in daily clinical practice rather than banal nevus (N:3,815). Early forms of malignant tumors such as mpd and bd which are a type of intraepidermal scc were included. Malignant forms of benign lesions with small number of data (such as dfsp, N:4, counterpart of df) were also included in the dataset. The inclusion of subclasses with the small number of samples aims to achieve a more complete taxonomy tree. These samples can be used to develop new algorithms and can be combined with other datasets to achieve higher number of samples to identify these rare diseases. The consensus of GG and SPY experts labeled all the benign images that did not have histology or further follow-up. GG (with a second Ph.D. in Basic Oncology) and SPY are two expert dermatologists with 20+ years of experience in dermatology.

### Baseline deep learning

Three Convolutional Neural Networks (CNNs) were trained to form a baseline for future studies: ResNet50^40^, Xception^41^, and InceptionResNetV2^42^ using Python (version 3.9.19) and TensorFlow (version 2.10.1). The images were scaled to 224 × 224 pixels and 299 × 299 pixels and stored as NumPy arrays. The images were augmented using these techniques: rotation (range: 45), zoom (range: 0.2), width shift (range: 0.2), horizontal and vertical flip. The models, pre-trained on the ImageNet dataset^43^, were trained on our dataset for 50 epochs without fine tuning and 200 epochs with fine tuning using training and test information in the metadata. The weighted accuracies of the training on the test set were 0.50, 0.59, and 0.58 for ResNet50, Xception, and InceptionResNetV2, respectively. All source codes are accessible at the link under the Code Availability section.

## Usage Notes

The dataset provided in this study represents the initial comprehensive collection of skin lesion data from Turkiye, serving as a distinctive and important asset for both the medical and machine learning fields. The launch of this dataset represents a notable progression, especially when considering the wide range of skin types, it addresses and the comprehensive classification of skin lesions it contains. The dataset is valuable due to the inclusion of various benign subclasses that closely resemble malignant tumors, distinguishing it from current collections and presenting a novel opportunity for the advancement of advanced diagnostic algorithms.

The absence of a rich sub-classification may result in misclassification. For instance, congenital nevus (which is not categorized as a separate subclass in the published datasets) can often be mistaken as a melanoma^36^. It is also important to know that the lesion is a congenital nevus as this lesion has a high risk of evolving into a melanoma^36^. We also included uncommon lesions that are difficult to collect or annotate such as Spitz/Reed nevus^44^. By using our detailed subclassification, this dataset enables researchers to develop and refine more reliable AI models capable of distinguishing benign from malignant ones, improving diagnostic accuracy and reducing the likelihood of misdiagnosis. The dataset includes 40 subclasses, allowing researchers to focus on more intelligent algorithms such as hierarchical learning^45^.

The dataset exhibits potential to serve as a fundamental component in the advancement of effective algorithms for identifying malignant skin lesions. The ongoing obstacle in dermatological diagnosis lies in the difficulty of differentiating between benign and malignant forms, particularly when they exhibit comparable visual characteristics. The incorporation of benign that exhibit similarities to malignant counterparts in this collection of data presents a potential for enhancing the precision of machine learning algorithms. Therefore, it is anticipated that this technology will facilitate progress in the early and accurate detection of diseases, potentially mitigating the occurrence of false positive results and ultimately enhancing patient prognoses.

The inclusion of the dataset originating from Türkiye contributes to the expansion of the worldwide data repository, which has historically been underrepresented in this research domain. The geographical specificity of this information is of great use to academics who seek to create diagnostic models that are both resilient and efficient across various ethnicities and geographic areas. Additionally, it functions as a valuable resource for doctors who aim to comprehend the diversity in lesion, which can be impacted by various factors such as regional environmental conditions and genetic predispositions.

Clinicians are encouraged to employ this dataset as a visual resource for the identification of skin lesions, while researchers are encouraged to utilize it for comparative analyses with other datasets specific to the location. This dataset is of particular utility to machine learning practitioners for the purposes of training and verifying classification algorithms. The comprehensive depiction of complex instances within the dataset offers a rigorous platform for evaluating algorithms intended to distinguish between benign and malignant tumors.

In brief, this dataset serves to enhance the existing body of data pertaining to skin lesions, while also paving the way for the development of sophisticated diagnostic instruments. We anticipate that this tool will be a helpful resource in enhancing the precision and effectiveness of skin cancer detection and diagnosis among various demographic groups.

In this dataset, there are 40 subgroup lesions. Brief descriptions of these subgroups presented below can be useful for AI researchers:

I. Melanocytic lesions: Originates from melanocytes, often pigmented, shades of brown, black, tan, blue. Reflecting varying concentrations and depth of melanin within the skin.

1. Benign: Non-cancerous melanocyte-origin moles, generally harmless.

- Dysplastic Nevus: Also known as atypical or Clark’s nevus. They are characterized by histologic features; they may appear small and banal clinically. Dysplastic nevi demonstrate the following clinical features: usually > 5 mm, irregular borders, some with a pigmented and erythematous rim, and variegated pigmentation with a mixture of pink, light, and dark brown colors^46,47^. This subclass contains compound nevus (cd) with acral (acd) and congenital (ccd) subclasses; junctional nevus (jd) with acral (ajd) and Spitz/Reed (srjd) subclasses; and recurrent nevus (rd).
- Banal Nevus: A banal nevus, commonly known as a common mole, is a typical, non-cancerous skin mole that usually appears as a small, regular, round, or oval spot. Brown, tan, or pink, and may be either flat or slightly raised. This subclass contains compound nevus (cb) with acral (acb), congenital (ccb), Miescher (mcb) subclasses; dermal nevus (db), with blue (bdb) subclass; and junctional nevus (jb) with acral (ajb) and congenital (cjb) subclasses.
- Lentigo: Pigmented and uniform. It is related to an increase in melanin expression by melanocytes. This subclass contains ink spot lentigo (isl), lentigo simplex (ls), and solar lentigo (sl).

i. Compound: Common mole, partially flat and partially elevated. Mix of dermal and epidermal melanocytes.
ii. Dermal: Common mole. Raised bump, flesh-colored or brown mole. Located within the dermis. Generally benign. Unna nevus is included.
iii. Junctional: Flat, typically dark or brown mole, located at the epidermis-dermis junction.
iv. Recurrent nevus: Incompletely removed nevus. Mole regrowth at previously excised site. Typically, benign but can be confused with melanoma.

A. Acral nevus: Mole on palms or soles. Usually benign and characterized by their distinctive location on the body where the skin is thicker.
B. Blue nevus: Deeply pigmented mole, blue-black color. Originates from deep dermal melanocytes. Usually, benign.
C. Congenital nevus: Mole present at birth that grows proportionally with the child. Sizes vary, potentially large. Increased melanoma risk later in life. Nevus spilus is included.
D. Ink Spot Lentigo (isl): Small, dark brown to black spots, resembling ink spots.
E. Lentigo Simplex (ls): Small, flat, and typically darker spot, a precursor to junctional nevus. Also known as simple lentigo.
F. Miescher nevus: Reveals a pseudo network around hair follicles. Commonly benign, typically brown, and found on the face.
G. Solar Lentigo (sl): Larger and typically found on sun-exposed areas, a precursor to seborrheic keratosis. While not primarily melanocytic, solar lentigo is influenced by melanocytic activity, and is intentionally classified under melanocytic-benign lentigo group.
H. Spitz/Reed nevus: A raised, pink, red or brown mole. Often mistaken for melanoma. Typically, benign in children. Sometimes, shows a starburst pattern.

2. Malign: Cancerous growth from melanocytes. High risk of spreading.

- Melanoma: Aggressive skin cancer. Arises from melanocytes. High risk of metastasis.

i. Acral nodular melanoma (anm): A nodular melanoma form on poles and soles. Amelanotic melanoma were also included.
ii. Acral lentiginous melanoma (alm): A subtype of melanoma. Lentiginous form on poles and soles.
iii. Lentigo maligna (lm): Larger, a precursor to lentigo maligna melanoma. A slow-growing skin cancer that appears as a flat, blotchy patch. It is an early form of melanoma, typically located on sun-exposed areas of the skin.
iv. Lentigo maligna melanoma (lmm): Various number of colors, particularly blue or black which often indicates that melanoma cells have reached deeper layers of skin.
v. Melanoma (mel): Melanoma can appear as a new dark spot on the skin or from an existing mole that changes in color, size, or feel. It can spread quickly to other parts of the body and is critical to treat early.

II. Nonmelanocytic lesions: Uncontrolled growth pattern from keratinocytes, fibroblasts, or vascular cells.

1. Benign: Non-cancerous growths from keratinocytes, fibroblasts, histiocytes or vascular cells.

- Keratinocytic:

i. Seborrheic keratosis (sk): A waxy, wart-like, often brown or black growth. Commonly has a rough texture. Stucco keratosis, a variant located on legs, is included.
ii. Lichenoid keratosis (lk): Inflammatory and often regressing on solar lentigo and seborrheic keratosis.

(b) Fibro-histiocytic:

i. Dermatofibroma (df): A firm, benign skin nodule. Common on the legs or arms. Typically, brownish, and harmless.

(c) Vascular:

i. Angiokeratoma (angk): Small, dark red to purple, raised lesions with a rough surface. Composed of dilated blood vessels. Typically seen on the lower body or genitals.
ii. Hemangioma (ha): Bright red, raised birthmark. Made of clustered blood vessels. Common in infants, usually fades.
iii. Lymphangioma (la): Rare, soft, often translucent mass. Appears as a swelling on the skin or mucous membranes. Typically seen in infants.
iv. Pyogenic granuloma (pg): Small, reddish, raised lesion. Prone to bleeding and rapid growth. Often appears after skin injury.
v. Spider angioma (sa): Small, red, spiderweb-like lesion with a central red dot and radiating capillaries. Common on the face and upper body.

2. Indeterminate: Not exactly categorized as benign or malignant. This does not imply that these lesions are malignant.

- Keratinocytic:

i. Actinic keratosis (ak): Rough, scaly skin patch. Sun-induced, precancerous with a variable risk of progression to malignancy. Common on sun-exposed areas.

3. Malign: Cancerous growths that can originate from keratinocytes, fibroblasts, histiocytes, and vascular cells.

- Keratinocytic:

i. Basal cell carcinoma (bcc): Appears as a shiny, pearly bump or a flat, scar-like lesion. Most common on sun-exposed areas. Slow growing and rarely metastasizes.
ii. Bowen’s disease (bd): Early form of scc. Appears as a persistent, red, scaly patch. Can resemble eczema or psoriasis. Often occurs on sun-exposed skin.
iii. Cutaneous horn (ch): Hard, protruding growth resembling an animal horn. Composed of compacted keratin mostly located on the face, ears, and other sun exposed areas. It may be associated with benign, premalignant, and malignant lesions. Only lesions with a malignant base is included.
iv. Mammary Paget’s Disease (mpd): An early form of scc and a type of breast cancer.
v. Squamous cell carcinoma (scc): Common, appears as a firm, red nodule, or a flat lesion with a scaly, crusted surface. Commonly develops in sun-exposed areas. Can be ulcerative and may bleed.

(b) Fibro-histiocytic:

i. Dermatofibrosarcoma protuberans (dfsp): Rare, appears as a deep-seated, firm lump under the skin. Often starts as a small, painless nodule that gradually enlarges. Typically develops on the trunk or limbs.

(c) Vascular:

i. Kaposi sarcoma (ks): Presents as purple, red, or brown blotches or tumors on the skin.

## Data Availability

Codes are available at https://github.com/abdurrahimyilmaz/derm12345
